# BMI1 promotes osteosarcoma proliferation and metastasis by repressing the transcription of SIK1

**DOI:** 10.1186/s12935-022-02552-8

**Published:** 2022-03-27

**Authors:** Qiang Wang, Yinghui Wu, Meng Lin, Gaigai Wang, Jinyan Liu, Min Xie, Bo Zheng, Cong Shen, Jun Shen

**Affiliations:** 1 Department of Orthopeadic Surgery, The Affiliated Suzhou Hospital of Nanjing Medical University, Gusu School, Nanjing Medical University; Suzhou Municipal Hospital, No.26, Daoqian Street, Suzhou, 215002 Jiangsu China; 2 State Key Laboratory of Reproductive Medicine, Department of Histology and Embryology, Nanjing Medical University, Nanjing, 211166 China; 3 State Key Laboratory of Reproductive Medicine, Center for Reproduction and Genetics, Suzhou Municipal Hospital; The Affiliated Suzhou Hospital of Nanjing Medical University, Gusu School, Nanjing Medical University, Suzhou, 215002 China; 4 Department of Breast and Thyroid Surgery, Suzhou Municipal Hospital; The Affiliated Suzhou Hospital of Nanjing Medical University, Gusu School, Nanjing Medical University, Suzhou, 215002 China

**Keywords:** Osteosarcoma(OS), BMI1, Proliferation, Metastasis, SIK1, Histone modification

## Abstract

**Background:**

Osteosarcoma (OS) is the most common malignant tumor of bone, and the clinical efficacy of current treatments and associated survival rates need to be further improved by employing novel therapeutic strategies. Although various studies have shown that BMI1 protein is universally upregulated in OS cells and tissues, its specific role and underlying mechanism have not yet been fully explored.

**Methods:**

Expression of BMI1 protein in OS cells was detected by western blot. The effect of BMI1 on proliferation and migration of OS cells (143B and U-2OS cell lines) was investigated in vitro using CCK-8, colony formation and transwell assays, and in vivo using subcutaneous tumorigenesis and lung metastasis assays in xenograft nude mice. Expression of epithelial–mesenchymal transition (EMT)-associated proteins was detected by immunofluorescence imaging. Bioinformatic analysis was performed using ENCODE databases to predict downstream targets of BMI1. SIK1 mRNA expression in osteosarcoma cells was detected by quantitative real-time reverse transcription PCR (qPCR). Chromatin immunoprecipitation-qPCR (ChIP-qPCR) was used to investigate expression of BMI1-associated, RING1B-associated, H2AK119ub-associated and H3K4me3-associated DNA at the putative binding region of BMI1 on the SIK1 promoter in OS cells.

**Results:**

Using both in vitro and in vivo experimental approaches, we found that BMI1 promotes OS cell proliferation and metastasis. The tumor suppressor SIK1 was identified as the direct target gene of BMI1 in OS cells. In vitro experiments demonstrated that SIK1 could inhibit proliferation and migration of OS cells. Inhibition of SIK1 largely rescued the altered phenotypes of BMI1-deficient OS cells. Mechanistically, we demonstrated that BMI1 directly binds to the promoter region of SIK1 in a complex with RING1B to promote monoubiquitination of histone H2A at lysine 119 (H2AK119ub) and inhibit H3K4 trimethylation (H3K4me3), resulting in inhibition of SIK1 transcription. We therefore suggest that BMI1 promotes OS cell proliferation and metastasis by inhibiting SIK1.

**Conclusions:**

Our results reveal a novel molecular mechanism of OS development promoted by BMI1 and provides a new potential target for OS treatment.

**Supplementary Information:**

The online version contains supplementary material available at 10.1186/s12935-022-02552-8.

## Background

Among primary malignant bone tumors, osteosarcoma (OS) is the most common, the annual incidence is 1 to 3/10,000,000 [1, 2]. The age distribution of patients presenting with OS is bimodal with peaks for children/adolescents (median age, 18 years) and elderly individuals aged 60 years and over [3]. The most common anatomical sites include the distal end of the femur, the proximal end of the tibia and the humerus metaphysis [2, 4]. OS tends to occur at the age of puberty at sites of maximum bone growth, suggesting a close association with rapid bone cell proliferation [2]. The cardinal symptom is pain caused by the tumor, which is produced by erosion and dissolution of bone tissue. About 20–30% of OS patients have metastatic lesions by the time they are diagnosed, and the lung is the most common site of distant metastasis [5]. Surgical resection and systematic chemotherapy have been the main therapeutic approaches since the 1970s, over which time therapeutic efficacy and survival rates have not improved dramatically [5, 6]. Therefore, there is an urgent need for novel therapeutic targets to increase survival.

Polycomb group (PcG) proteins consist of polycomb repressive complex 1 (PRC1) and polycomb repressive complex 2 (PRC2) [7]. The proto-oncogene B lymphoma Mo-MLV insertion region 1 (BMI1), a key component of PRC1, is a transcriptional repressor that has been shown to be involved in tumorigenesis, cell cycle regulation and stem cell maintenance [8–12]. BMI1 acts as an oncogene by forming complexes with other members of the PcG to inhibit expression of tumor suppressor genes [13]. Previous reports indicated that BMI1 is associated with the occurrence, progression, and prognosis of multiple tumor types, including bladder cancer [12, 14], non-small cell lung cancer [15], colon carcinoma [16], colitis-associated cancer [17], breast cancer [18], glioblastoma [19], and hepatocellular carcinoma [8]. BMI1 has also been studied in OS tissues and cell lines. Data from 3 independent studies showed that BMI1 is ubiquitously upregulated in OS tissues, demonstrating a positive correlation between BMI1 and OS progression [7, 20, 21]. However, Sasaki et al. [22] reported that knockdown of BMI1 in OS cells does not affect their phenotype. Other studies have shown that BMI1 can promote proliferation, migration and chemoresistance of OS cells [20, 23]. Therefore, the role of BMI1 in regulating OS tumor cells remains controversial and requires further investigation.

Salt-inducible kinases (SIKs) are a subfamily of the AMP-activated protein kinase (AMPK) family, initially named SIKs because their expression in the adrenal gland is related to dietary salt intake [24]. SIKs include SIK1, SIK2 and SIK3 [25]. Although SIKs share characteristic structural domains, expression of SIK1 is regulated by external stimuli, which is in contrast to constitutive expression of SIK2 and SIK3 [24]. Previous studies identified SIK1 as a potent tumor suppressor [26]. Reduced LKB1-SIK1 signaling promotes epithelial–mesenchymal transition (EMT) and resistance to radiation treatment of tumor cells in non-small cell lung cancer [27, 28]. Several studies revealed that the expression of SIK1 is downregulated in ovarian cancer and hepatocellular carcinoma [29, 30]. In a hepatocellular carcinoma xenograft tumor model, overexpression of SIK1 can significantly inhibit EMT, tumor growth and lung metastasis [30].

The aim of this study was to explore the function of BMI1 during OS proliferation and metastasis. In this study, we predicted the potential targets of BMI1 in OS cell lines (143B and U2OS) by ChIP-seq data and gene ontology (GO) analysis. Among the numerous potential downstream targets of BMI1, we focused on SIK1. However, no study of the correlation between BMI1 and SIK1 has been reported. As such, we further investigated the mechanism by which BMI1 targets SIK1 in OS cells. Our data demonstrate that BMI1 modulates OS progression by epigenetically repressing SIK1 expression, which may provide new insights into the regulatory mechanism of BMI1 in OS progression and help to develop novel strategies for OS treatment.

## Methods

### Cell culture, reagents, siRNA and transfection

OS cell lines (143B and U-2OS) were obtained from Zhong Qiao Xin Zhou Biotechnology Co., Ltd (Shanghai, China). U-2OS cells were maintained in Dulbecco’s Modified Eagle’s Medium (DMEM) with 10% fetal bovine serum (FBS) (Thermo Scientific, Waltham, MA, United States) and 100 U/ml penicillin and 100 mg/ml streptomycin (Invitrogen). 143B cells were maintained in Eagle’s Minimum Essential Medium (EMEM), which contains Earle’s salts, l-glutamine, non-essential aminoacids, NaHCO3, 10% FBS, 100 U/ml penicillin and 100 mg/ml streptomycin, and 5-bromine-2-deoxyuridine. All cells were cultured in a 5% CO_2_ humidification incubator at 37 ℃. PTC-209, a specific BMI1 inhibitor [8, 31], was obtained from Selleck (Houston, TX, USA). DMSO for dissolving PTC-209 was purchased from Thermo Fisher Scientific (Waltham, MA, USA).

When cells were 60–70% confluent, we used Lipofectamine 2000 (Invitrogen, USA) to transfect OS cells with BMI1, SIK1 siRNAs and the negative control siRNA (si-NC). Functional assays were conducted after 48 h. Nucleotide sequences included:

- siBMI1 #1 (5′CCUGGAGACCAGCAAGUAUTT-3′),
- siBMI1 #2 (5′CCAGAUUGAUGUCAUGUAUTT-3′),
- siSIK1 #1 (5′GGUUCAGCUGAUGAAGCUUTT-3′),
- siSIK1 #2 (5′GGAACCAGCUCUGACAGUUTT-3′),
- siSIK1 #3 (5′GGAGUACUGUCACGACCAUTT-3′), and
- siNC ( 5′UUCUCCGAACGUGUCAGGUTT-3′ ).

### Western blot

Total protein was extracted from OS cells by rapid tissue lysate RIPA Lysis Buffer, which contained 1% PMSF (phenylmethylsulfonyl fluoride) as a protease inhibitor. Protein levels were quantified using a bicinchoninic acid (Beyotime Biotechnology) kit. Protein was separated by SDS-PAGE (sodium dodecyl sulfate-polyacrylamide gel electrophoresis) and transferred onto polyvinylidene difluoride (PVDF) membranes. The PVDF membranes were blocked with 5% skim milk for 1 h, and then incubated with primary antibody (Additional file 1: Table S1) at 4 ℃ overnight. Secondary antibodies combined with horseradish peroxidase at room temperature were incubated for 1 h. Western fluorescent detection reagent was applied evenly to the PVDF membranes. Imaging software (Image-Pro Plus Software 6.0) was then used to analyze the bands.

### Cell proliferation assays

When OS cells were at about 60–70% confluence, they were treated with the indicated concentrations of PTC-209 for 48 h. Cells were seeded into 96-well plates, 2500 in each well. Cell Counting Kit-8 kit (Beyotime Biotechnology) was used to detect cell viability every 24 h.

In the colony formation assay, cells were inoculated into 6-well plates, 1000 cells in each well, and then cultured in complete medium for 14 days, during which the medium was changed every 5 days. After 14 days, culture plates were washed twice with phosphate-buffered saline (PBS) solution, and cells were fixed with methanol for 15 min and stained with 0.1% crystal violet (Beyotime Biotechnology) for 15 min. Cell colonies were photographed and counted.

### Cell migration and invasion assays

Cells were treated with the indicated concentrations of PTC-209 for 48 h. Transwell assays were carried out using migration chambers with 8 μm membranes (Millipore, Billerica, MA, USA) placed in a 24-well plate. 25,000 cells in 300 µl serum-free medium were inoculated into the upper chamber while 700 µl complete medium was added to the lower chamber. After 48 h, the cells on the upper surface of the membrane were wiped off with cotton swabs, while those that had migrated to the lower surface of the membrane were fixed with methanol for 15 min and stained with 0.1% crystal violet for 15 min. After the chamber was gently rinsed twice in PBS, 5 visual fields were randomly selected under the microscope for cell counting.

The invasion assays were performed using migration chambers separated by polycarbonate filters with 8-µm pore diameter and pre‐coated Matrigel (Corning, Corning, NY). Equal numbers of 143B or U-2OS cells (50,000 cells) in serum free DMEM were added to the upper chamber. The lower chamber was filled with DMEM medium containing 10% FBS or EMEM medium for inducing cell migration, and plates were incubated for 48 h. Cells that traversed the membrane filter to the lower surface were fixed by methanol for 15 min and stained with 0.1% crystal violet for 15 min. After the chamber was gently rinsed twice in PBS, five randomly selected views were imaged and counted in each well.

### In vivo assays

4-week-old female athymic BALB/c nude mice were fed under specific pathogen-free conditions and treated according to protocols approved by the Committee on the Ethics of Animal Experiments of Nanjing Medical University. Animal use was approved by the Animal Ethical and Welfare Committee (AEWC) of Nanjing Medical University (Permit Number: IACUC-2004020). 143B cells were used for in vivo assays. Cells in the experimental group were treated with PTC-209 for 48 h, while cells in the control group were treated with DMSO.

For an in vivo cell proliferation assay, after washing the culture plates with PBS, cells were digested with trypsin and resuspended in PBS at a concentration of 3 × 10^7^ cells/ml. Each nude mouse was subcutaneously injected with 150 µl of suspended cells. The bilateral sides of each nude mouse were injected with treatment and control cells, respectively. After 16 days, the mice were anesthetized via inhalation of carbon dioxide. The mice lost consciousness rapidly and were sacrificed by cervical dislocation, according to the American Veterinary Medical Association (AVMA) Guidelines for the Euthanasia of Animals (2020 Edition). In short, the spinal cords were disconnected from the brain with force and speed. The subcutaneous tumors were removed, weighed and photographed, and tumor volume (0.5 × length × width^2^) was calculated.

For an in vivo cell metastasis assay, cells were resuspended at a concentration of 6 × 10^7^ cells/ml. Then each tail vein was injected with 100 µl of the suspended cells. 2 months later, the mice were euthanized and the lungs were removed and photographed. Metastatic tumor nodules were counted and evaluated by immunofluorescence.

### Histological analysis

Fresh subcutaneous tumor tissue and lung metastasis tissue were fixed with 4% paraformaldehyde for 48 h. After being serially dehydrated with ethanol and hyalinized with xylene, the tissues were embedded in paraffin, cut into 6 μm sections, deparaffinized with xylene and rehydrated with high to low concentrations of ethanol.

Samples were stained with hematoxylin and eosin (H&E) and dehydrated with low to high concentrations of ethanol. After being hyalinized with xylene and mounted with neutral balsam, the samples were observed and photographed under a microscope (Axioskop 2 plus, Zeiss).

For immunofluorescence, antigen was prepared in 10 mmol/L sodium citrate buffer (pH6.0) for 20 min. The sections were then blocked with 5% bovine serum albumin (BSA, w/v; Sunshine, Nanjing, China) and incubated with primary antibodies (Additional file 1: Table S1) at 4 °C overnight. After washing with PBS, the sections were incubated with Alexa-Fluor secondary antibodies (Thermo Scientific, Waltham, MA, USA). Cell nuclei were stained with 5 µg/ml 4’,6-diamidino-2-phenylindole (DAPI, Beyotime Institute of Biotechnology) for 10 min. All samples were viewed in a confocal laser microscope (Zeiss LSM710, Carl Zeiss, Oberkochen, Germany).

### RNA extraction and quantitative real-time reverse transcription-PCR

RNA extraction and quantitative real-time reverse transcription-PCR (qRT-PCR) was conducted as previously described [32]. Briefly, we used Trizol reagent to extract RNA from cells, and then reversibly transcribed the RNA to cDNA with a reverse transcription kit. SYBR Premix Ex Taq (Takara, Dalian, China) was used for qRT-PCR assays, which were conducted on an Applied Biosystems 7500 Real-Time PCR System. Primers used included the following:

- human SIK1, forward 5′-CACTCACCGCGCCATGTAT-3′
- and reverse 5′-GCTGACAGGGAGCAGAACAG-3′;
- human TWIST1, forward 5′- TCAAAGAAACAGGGCGTGGG-3′
- and reverse 5′- GCACGACCTCTTGAGAATGC-3′;
- human ZEB1, forward 5′- GCTGTTTCAAGATGTTTCCTTCCA-3′
- and reverse 5′ ACAGCGGCAACAGCTCAATA-3′;
- human SNAI1, forward 5′- AAGATGCACATCCGAAGCCA-3′.
- and reverse 5′- CATTCGGGAGAAGGTCCGAG-3′;
- human E-cadherin, forward 5′- CACCACGGGCTTGGATTTTG-3′
- and reverse 5′- AAATGTGTCTGGCTCCTGGG-3’;
- human N-cadherin, forward 5′- CCCTGCTTTCATTCTGACATACC-3′
- and reverse 5′- CTGCCACTTGCCACTTTTCC-3′;
- human Vimentin, forward 5′- AAACTTAGGGGCGCTCTTGT-3′
- and reverse 5′- GAGGGCTCCTAGCGGTTTAG-3′;
- human 18srRNA, forward 5′-AAACGGCTACCACATCCAAG-3′
- and reverse 5′-CCTCCAATGGATCCTCGTTA-3′.

### Chromatin immunoprecipitation assays

The chromatin immunoprecipitation-qPCR (ChIP-qPCR) assay was used to explore the underlying mechanism by which BMI1 acts on SIK1. An EZ-ChIP Kit (Millipore, Billerica, MA, United States) was used to conduct the ChIP assay. In brief, 143B and U-2OS cells were harvested, washed, and cross-linked by 1% formaldehyde. Then the cells were sonicated (Branson Sonicator 250) to shear DNA into ∼500-bp fragments. Next, antibodies (Additional file 1: Table S1) were reacted with the chromatin DNA–protein complexes. About 10% of the starting material was used. Immunoprecipitated DNA was analyzed by real-time qPCR using the following primers:

human SIK1 promoter, forward 5′- TCAGAGCCGCCAGTCTCTTG-3′

and reverse 5′-TTGACGTCGCTTTGACGCC-3′;

human GAPDH gene, forward 5′-AACCCAAACTAACAGTTGTCCCAA-3′

and reverse 5′-ACTCCTTGGAGGCCATGTAGG-3′.

### Statistical analysis

Statistical analyses were performed using GraphPad Prism software. All values from at least 3 independent experiments were expressed as mean ± standard deviation (SD). Unpaired Student’s t-test (2 groups) or one-way ANOVA (multiple groups) were used to analyze differences between groups. P values < 0.05 were considered statistically significant.

## Results

### BMI1 promotes proliferation, migration and invasion of OS cells in vitro

**Fig. 1:**
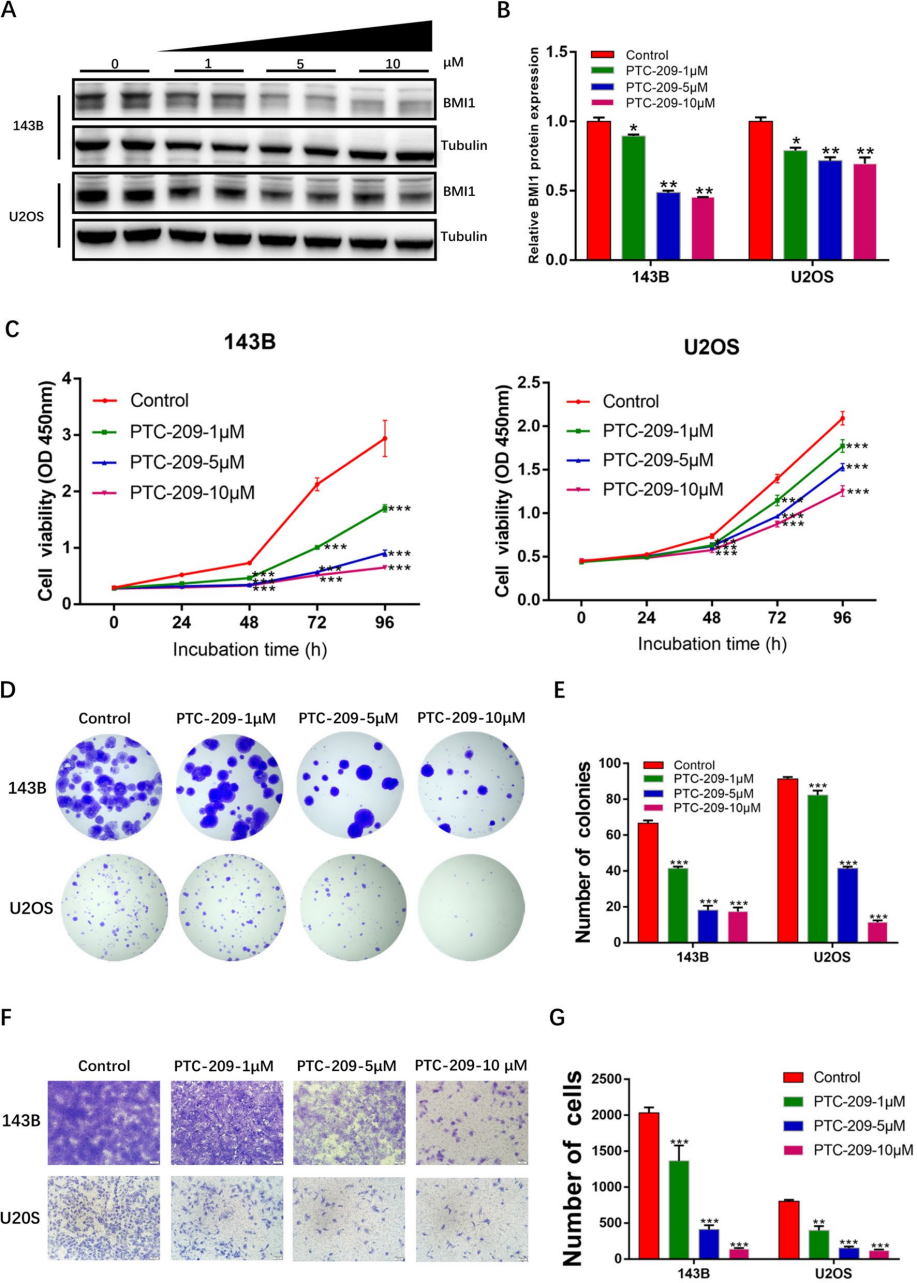
BMI1 affects OS cells proliferation and migration in a dose-dependent way. **A** OS cells were treated with PTC-209 for 48 h, followed by western blot analysis. Tubulin was used as an internal control. Sample size = 3. **B** Quantification of **A**. **C** After OS cells were treated with the indicated doses of PTC-209 for 48 h, the proliferative viability of OS cells was detected using CCK-8 assay. Sample size = 6. **D** Colony formation assays were used to detect the colony forming ability of OS cells after treated with the indicated doses of PTC-209 for 48 h. Sample size = 3. **E** Quantification of **D**. **F** After treated with the indicated doses of PTC-209 for 48 h, transwell assays were conducted to investigate migratory abilities of OS cells. Sample size = 3. **G** Quantification of **F**. *p < 0.05, **p < 0.01, ***p < 0.001, compared with control

PTC-209, a BMI1 specific inhibitor [8, 32], was used to downregulate BMI1 expression in both 143B and U2OS cell lines. A significant decrease in BMI1 expression was observed following 48 h of treatment of OS cells with PTC-209 (Fig. 1A, B). Next, CCK-8 assays showed that proliferation of OS cells was inhibited by PTC-209 in a dose-dependent manner (Fig. 1C). Colony formation assays showed a marked decrease in the ability of cells to form colonies (Fig. 1D, E). Transwell assays were utilized to assess the effect of BMI1 on the migrational ability of OS cells, and a loss of BMI1 was shown to reduce the number of the migrational OS cells (Fig. 1F, G).

In addtion, we also used BMI1-siRNA to inhibit the expression of BMI1 in OS cells (143B and U2OS). We observed that BMI1-siRNA treatment led to a substantial decrease in cell proliferative viability (Additional file 2: Fig. S1A-C). To estimate the migrational and invasive ability of OS cells, we performed transwell and invasion assays, the results showed a significant reduction in both migrational (Additional file 2: Fig. S1D,E) and invasive ability (Additional file 2: Fig. S1F,G) of BMI1-siRNA treated OS cells.

Cancer cell migration and invasion are associated with aberrant induction of EMT [33]. When EMT occurs, epithelial markers (such as Ecadherin) are attenuated while mesenchymal markers (such as Ncadherin and vimentin) are upregulated [34, 35]. Immunofluorescence staining showed higher E-cadherin staining and lower N-cadherin and Vimentin staining in the OS cells of the PTC-209 treated group as compared with the control group (Additional file 2: Fig. S2A-B). Additionally, we also tested EMT markers (E-cadherin, N-cadherin, and Vimentin) and the associated transcription factors, such as TWIST1 [36], ZEB1 [37] and SNAIL1 [38] in OS cells via qRT-PCR (Additional file 2: Fig. S2C). We observed that the mRNA expression of E-cadherin was significantly decreased while the mRNA expressions of N-cadherin, Vimentin, TWIST1, ZEB1 and SNAIL1 were increased in the PTC-209 treated OS cells, when compared with the control group.

The above results strongly suggested that BMI1 is required for the proliferative, migrational and invasive abilities of OS cells.

### BMI1 promotes tumorigenesis of OS cells in vivo

**Fig. 2:**
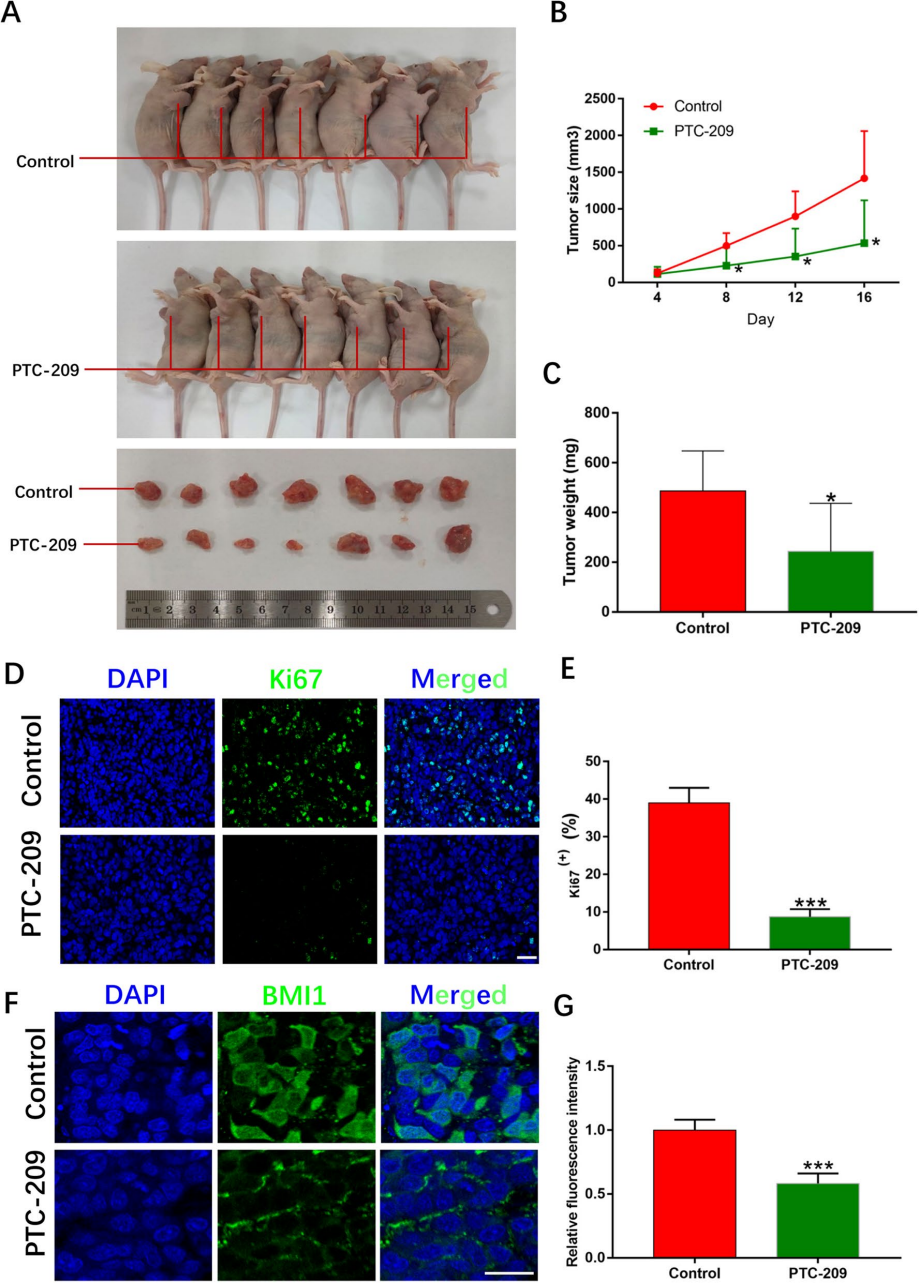
BMI1 affects OS cells tumorigenesis in vivo. **A** OS cells treated with 10µM PTC-209 for 48 h and control group were injected subcutaneously into the armpits of nude mice. The tumor volume of the PTC-209 group was significantly larger than that of the control group. Sample size = 7. **B** Tumor sizes were represented as means of tumor sizes ± SD. **C** Tumor weights were represented as means of tumor weights ± SD. **D** Immunofluorescence staining of Ki67 in control and PTC-209 treated group. Sample size = 3. Scale bar: 20 μm. **E** Quantification of **D**. **F** Immunofluorescence of BMI1 in control and PTC-209 treated group. Sample size = 3. Scale bar: 20 μm. **G** Quantification of **F**. *p < 0.05, ***p < 0.001, compared with control

To determine the ability of OS cells to form tumors in vivo, we subcutaneously injected BMI1 knockdown and control OS cells (143B) into the armpits of nude mice. After 16 days, the tumor volumes of the PTC-209 group were significantly smaller than those of the control group (Fig. 2A, B). Moreover, tumor weight of the PTC-209 group at the end of the experiment was markedly lower than the control group (Fig. 2C). The reduced tumor growth was also confirmed by Ki67 immunofluorescence staining as the proportions of Ki67-positive cells were significantly decreased in the PTC-209-treated group (Fig. 2D, E). Ulteriore analysis showed that BMI1 expression was significantly lessened in tumor tissues formed from PTC-209 treated cells (Fig. 2F, G).

### BMI1 promotes metastasis of OS cells in vivo

**Fig. 3:**
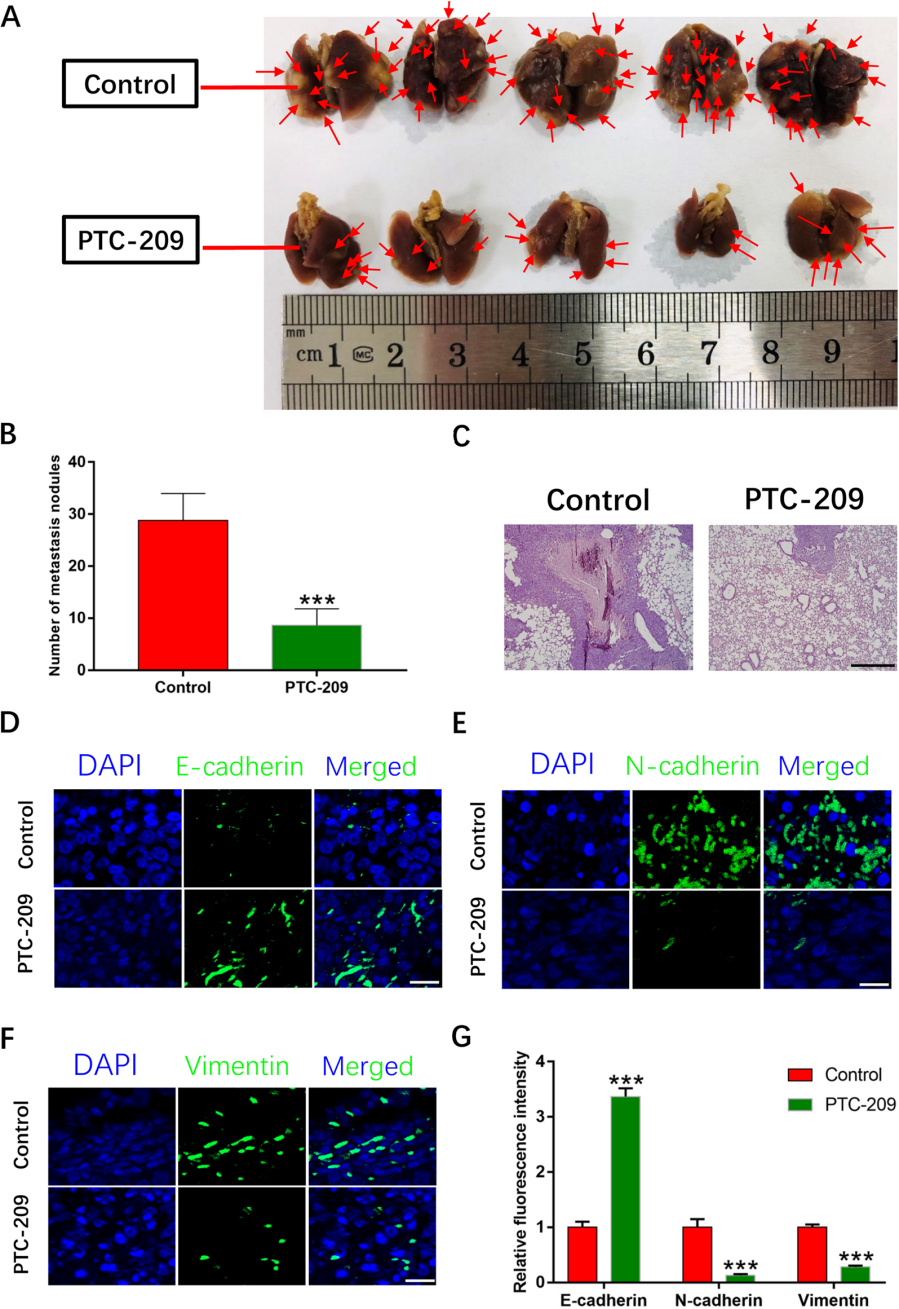
BMI1 affects 143B OS cells metastasis in vivo. **A** Cells treated with 10µM PTC-209 for 48 h and control cells were injected into the tail vein of nude mice. Lung tissues were took out and photographed. Sample size = 5. **B** Counting the number of lung nodules. **C** H&E staining was performed on paraffin sections of lung tissues. Sample size = 3. Scale bar: 500 μm. **D-F** Immunofluorescence staining of E-cadherin, N-cadherin, and Vimentin in control and PTC-209 treated group. Sample size = 3. Scale bar: 20 μm. **G** Quantification of **D–F**. ***p < 0.001, compared with control

Metastasis is the migration of cancer cells from the original tumor to distant sites, and is considered to be the most serious feature of advanced malignancies [39]. To investigate the in vivo metastatic ability of OS cells, BMI1 knockdown and control OS cells were injected into the tail veins of nude mice. After 2 months, lung tissues were collected and examined for metastatic nodules. The number of metastatic lung nodules in the BMI1 knockdown group was significantly less than in the control group (Fig. 3A, B). H&E staining provide a better depiction of lung metastatic nodules (Fig. 3C). Evaluation of the cadherins and vimentin by immunofluorescence staining (Fig. 3D–G) and western blot (Additional file 2: Fig. S2D, E) in OS tissues showed a significant increase in expression of E-cadherin and a decrease in expression of N-cadherin and vimentin in the PTC-209 group as compared with the control group. Furthermore, we also tested EMT markers and the associated transcription factors in the metastatic nodules via qRT-PCR (Additional file 2: Fig. S2F). qRT-PCR results from in vivo (Additional file 2: Fig. S2F) and in vitro (Additional file 2: Fig. S2C) were highly consistent.

Collectively, the above data demonstrated that BMI1 act as an oncogene promoting OS cell proliferation and metastasis.

### BMI1 represses transcription of SIK1

**Fig. 4:**
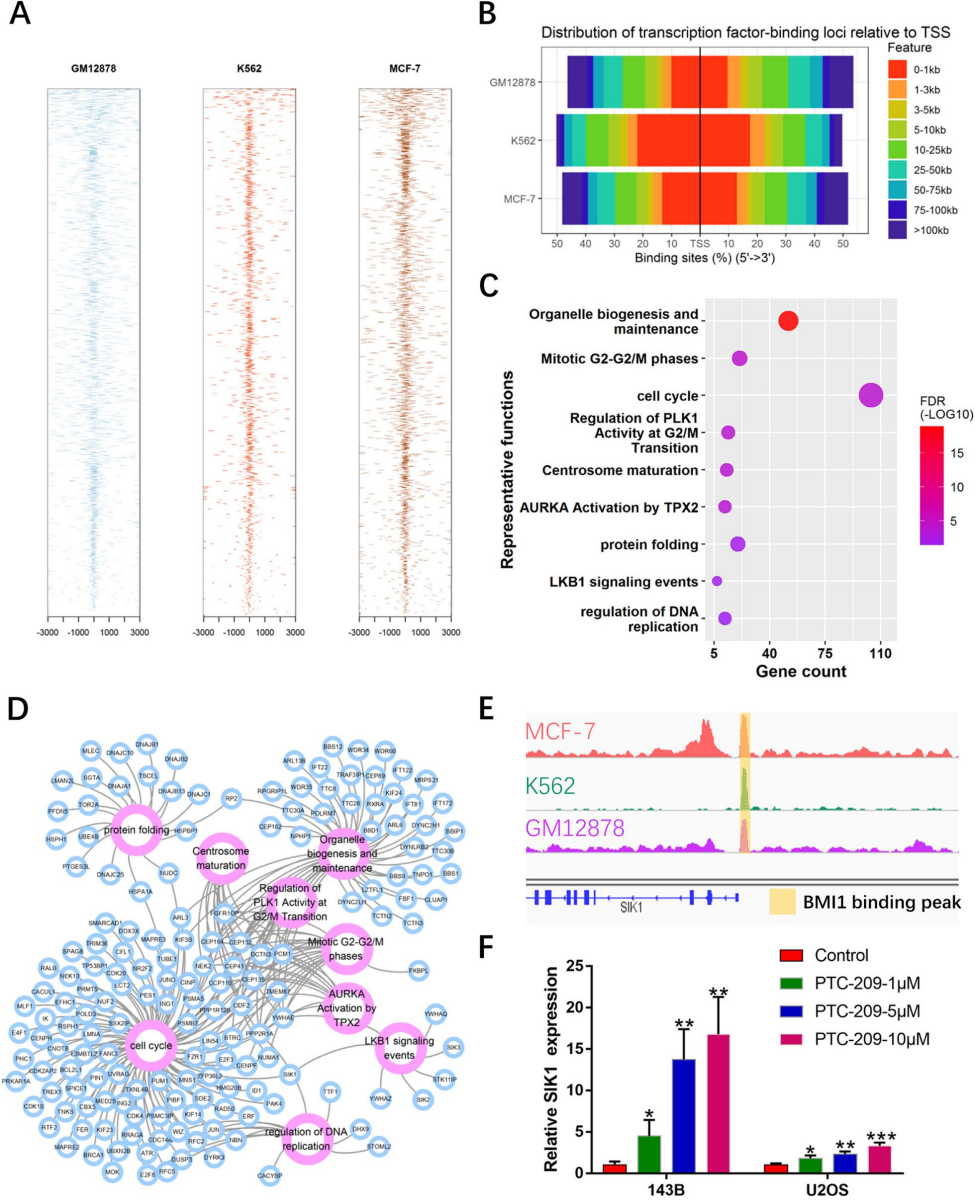
SIK1 is a potential downstream gene of BMI1. **A**, **B** Distribution of transcription factor-binding loci relative to Transcription Start Sites (TSS) in the indicated cell lines. **C** Bubble plot of functional enrichment: the color represents the p value, and the circle size represents the number of genes. **D** Network diagram of functional enrichment: purple circles represent function, blue circles represent gene, lines represent the presence of a relationship between gene and function. **E** A common transcription factor binding peak for BMI1 in the promoter region of SIK1 in the indicated cell lines. **F** After treated with the indicated doses of PTC-209, the relative expression of SIK1 in OS cells was detected by qRT-PCR. Sample size = 3. *p < 0.05, **p < 0.01, ***p < 0.001, compared with control

Publicly available ENCODE datasets (www.encodeproject.org) were utilized to predict binding sites for the transcription factor BMI1. Bioinformatic analysis of publicly available BMI1 ChIP-seq data for the human MCF-7 cell line (ENCSR967YYJ), K562 cell line (ENCSR782WRO) and GM12878 cell line (ENCSR469WII) revealed an enriched distribution of BMI1 binding sites from − 1 kb to + 1 kb relative to the Transcription Start Site (TSS) (Fig. 4A, B). Based on the common enrichment binding sites of BMI1 in these 3 cell lines, we performed GO analysis and found that the direct targets of BMI1 are significantly enriched for organelle biogenesis and maintenance, mitotic G2-G2/M phases, cell cycle control, regulation of PLK1 activity at the G2/M transition, centrosome maturation, AURKA activation by TPX2, protein folding, LKB1 signaling events and regulation of DNA replication (Fig. 4C, D). Among these target genes, we focused on a tumor suppressor, SIK1, and sought to determine whether BMI1 directly regulates SIK1 transcription.

**Fig. 5:**
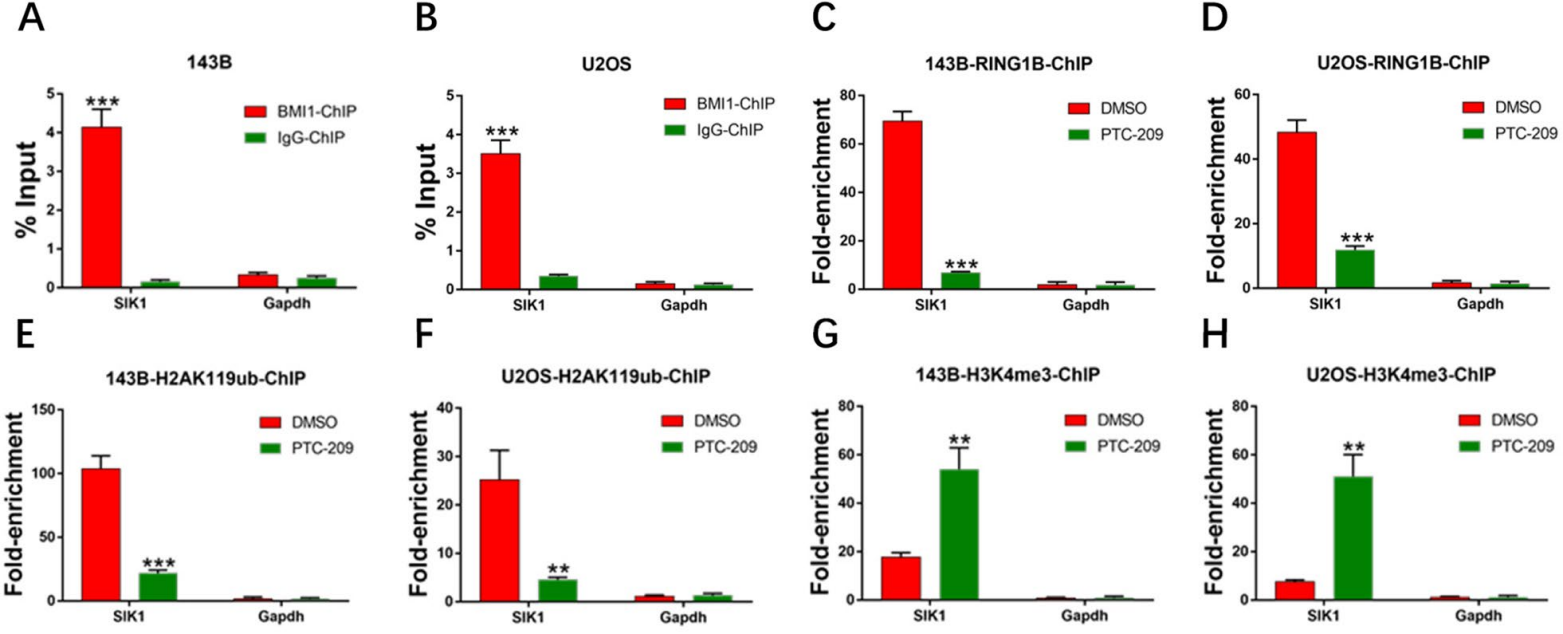
BMI1 inhibition alters the chromatin status at the BMI1 binding loci in the SIK1 promoter region. **A**, **B** ChIP-qPCR of BMI1-associated DNA sequences from the BMI1-binding region of the SIK1 promoter in OS cells. The Gapdh gene was used as a negative control. Sample size = 3. **C–H** ChIP-qPCR of RING1B-associated, **C**, **D** H2AK119ub-associated, **E**, **F** and H3K4me3-associated, **G**,**H** DNA sequences in the BMI1-binding region of the SIK1 promoter in DMSO-treated or PTC-209-treated OS cells. The y-axis represents enrichment relative to IgG controls. Sample size = 3. **A**, **B** ***p < 0.001, compared with IgG-ChIP. **C–H** **p < 0.01, ***p < 0.001, compared with DMSO

We observed a common binding site for BMI1 in the promoter region of SIK1 in the 3 cell lines referred to above (Fig. 4E). Moreover, mRNA expression of SIK1 increased in PTC-209-treated OS cells (Fig. 4F) in a dose-dependent manner. In the nucleus, BMI1 and RING1B (RING2) are the core components of PRC1, and they are both involved in monoubiquitination of lysine 119 of histone H2A, resulting in silencing of target genes [40, 41]. As such, we performed a ChIP-qPCR assay in OS cells to measure the effects of expression of BMI1, RING1B, H2AK119ub and H3K4me3 on the promoter region of SIK1.The BMI1 binding peak sequence of the promoter region of SIK1 was precipitated with BMI1 (Fig. 5 A, B). When BMI1 was significantly knocked down by PTC-209, precipitation of RING1B and H2AK119ub at the BMI1 binding peak sequence was greatly reduced (Fig. 5C–F), suggesting that function of the PRC1 complex was disrupted. Recently, we and other groups found that H3K4me3 contributes to derepression of BMI1 target genes [42]. Indeed, precipitation of H3k4me3 at the BMI1 binding peak region in the SIK1 promoter was obviously up-regulated in PTC-209-treated OS cells (Fig. 5G, H). In summary, we demonstrated that BMI1 represses SIK1 transcription by forming a complex with RING1B that binds to the promoter region of SIK1.

### Knockdown of SIK1 can promote proliferation and migration of OS cells

**Fig. 6:**
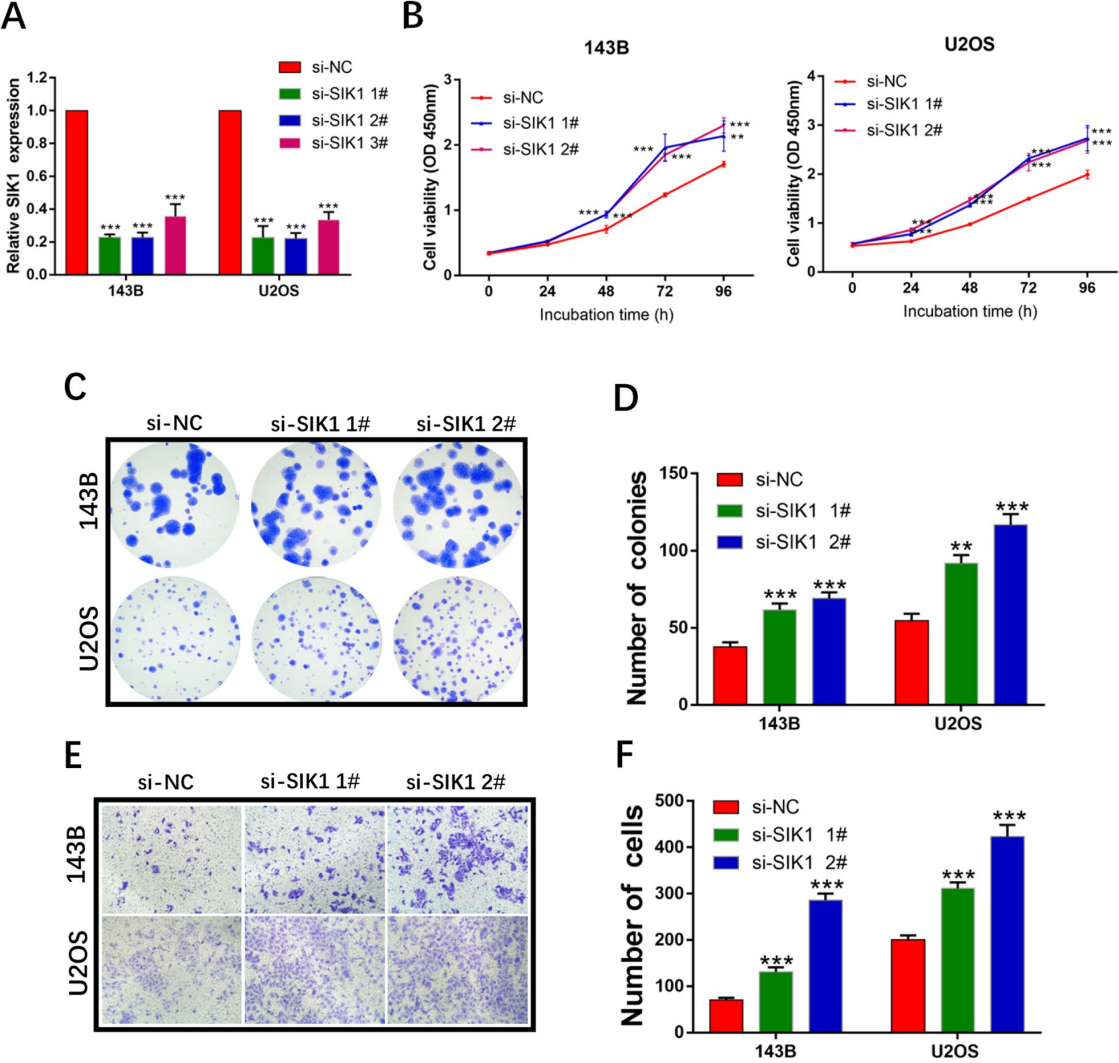
SIK1 functions as a tumor suppressor in OS cells. **A** Relative expression levels of SIK1 in OS cells transfected with si-NC or si-SIK1 1#, 2#, 3#, were tested by qRT-PCR. Sample size = 3. **B** CCK-8 assays were performed in OS cells transfected with si-NC or si-SIK1 1#, 2#. Sample size = 6. **C** Colony formation assays were used to determine the proliferative ability of OS cells transfected with si-NC or si-SIK1 1#, 2#. Sample size = 3. **D** Quantification of **C**. **E** Transwell assays in OS cells transfected with si-NC or si-SIK1 1#, 2#. Sample size = 3. **F** Quantification of **E.** **p < 0.01, ***p < 0.001, compared with si-NC

To explore whether SIK1 functions as a tumor suppressor in OS cells, we decreased SIK1 expression by transfecting three different siRNAs, siRNA #1, siRNA #2, and siRNA #3, into OS cells. As shown in Fig. 6A, siRNA #1 and siRNA #2, which were selected for subsequent functional assays, were more effective in silencing SIK1 compared with the negative control. After transfecting the cells with siRNA #1 and siRNA #2, CCK-8 assays (Fig. 6B) and colony assays (Fig. 6C, D) showed that OS cell proliferation was significantly enhanced when the expression of SIK1 was knocked down. Next, migration of OS cells was tested using transwell assays (Fig. 6E, F). As expected, more cells crossed the chamber membrane in the SIK1- siRNA #1 and siRNA #2 groups than in the negative control group. Taken together, the above results illustrate that SIK1 functions as a tumor suppressor in OS cells.

### Knockdown of SIK1 can restore proliferation and migration to BMI1-deficient OS cells

**Fig. 7:**
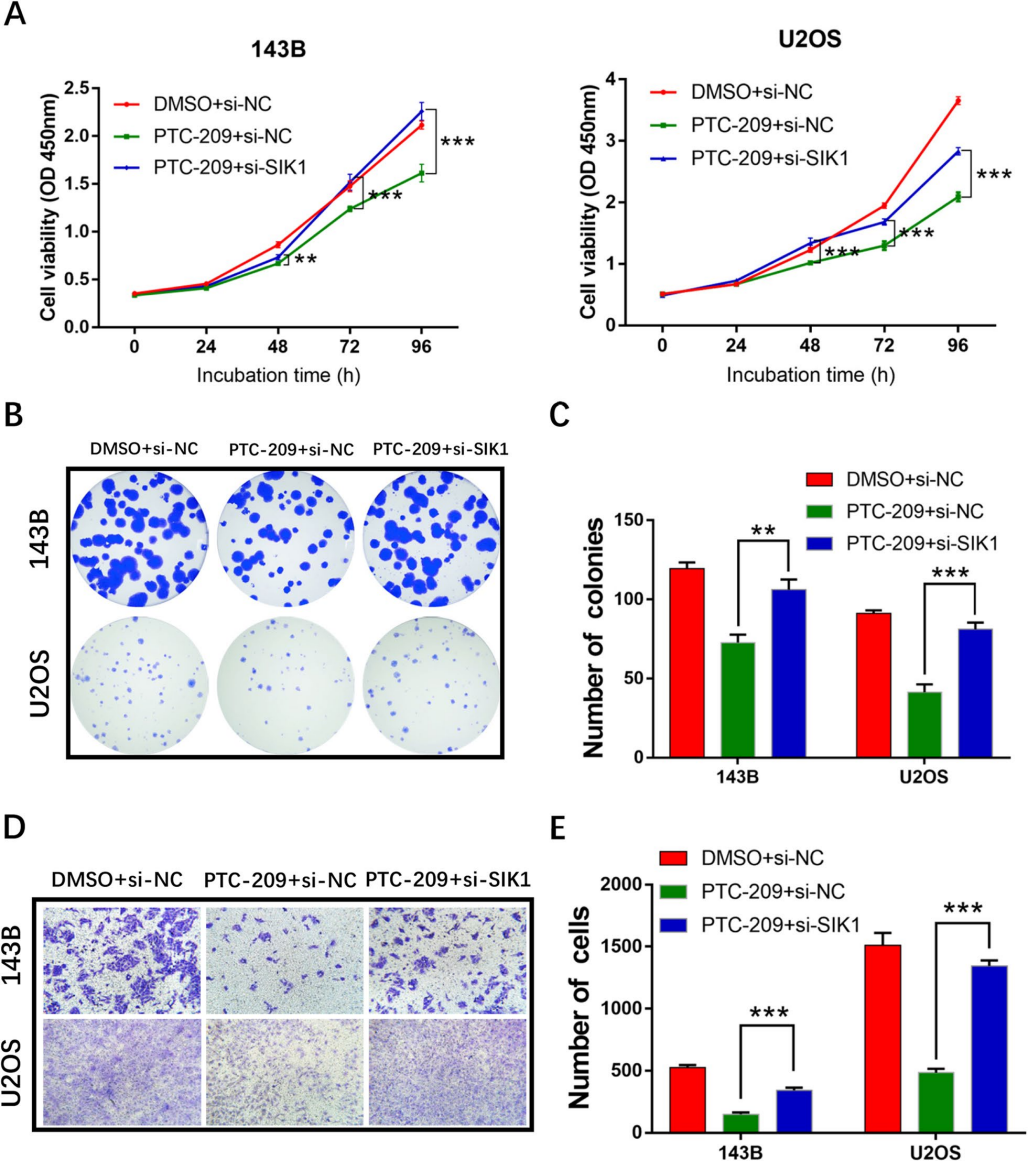
Downregulation of SIK1 can restore the altered phenotypes caused by the loss of BMI1. **A** After treated with the indicated way for 48 h, the proliferative ability of OS cells was detected using CCK-8 assays. Sample size = 6. **B** After treated with the indicated way for 48 h, colony formation assays were conducted in OS cells. Sample size = 3. **C** Quantification of **B**. **D** After treated with the indicated way for 48 h, transwell assays were performed to determine the migration ability of OS cells. Sample size = 3. **E** Quantification of **D**. **p < 0.01, ***p < 0.001, compared with PTC-209 + si-NC

To further test whether the aberrant elevation of SIK1 contributes to the defective tumorigenesis observed in PTC-209-treated OS cells, we downregulated SIK1 expression by siRNA-mediated gene silencing. The results showed that OS cells treated with both si-SIK1 and PTC-209 exhibited enhanced cellular proliferation (Fig. 7A–C) and migration (Fig. 7D, E) compared with OS cells treated with PTC-209 alone. Taken together, suppression of SIK1 expression can rescue the tumor suppressive effects of BMI1 loss.

**Fig. 8:**
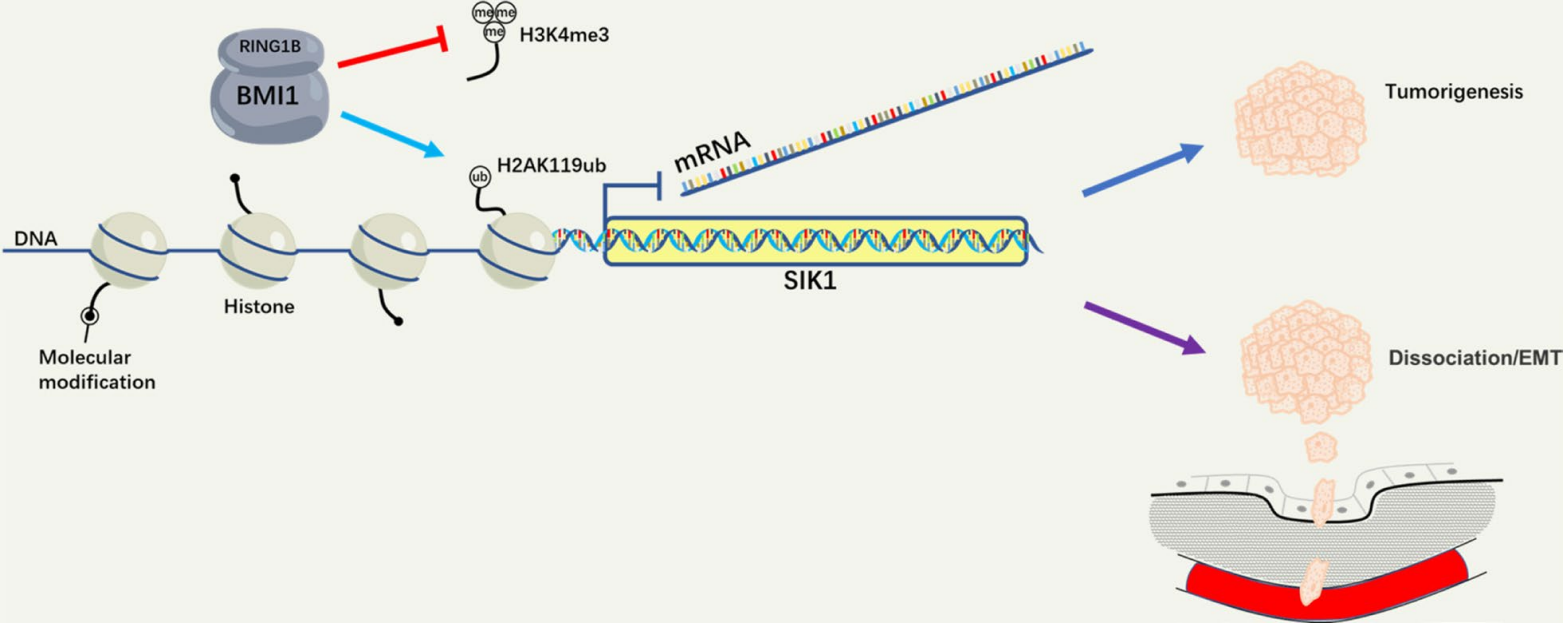
Schematic illustration of the working model for the role of BMI1 in OS cells

All of the above in vitro and in vivo results suggest that BMI1 functions as a proto-oncogene in OS cells by assembling PRC1 to regulate chromatin accessibility and repress transcription of SIK1 (Fig. 8).

## Discussion

Osteosarcoma, the most common malignant tumor of bone, is characterized by rapid progression, high metastatic potential and poor prognosis [43]. OS occurs via a multifactorial and multistep process, and its pathogenesis remains unclear and requires further investigation [44]. Many oncogenes or tumor suppressor genes have been reported to be involved in the progression of OS, including the oncogene BMI1 [20, 45–47]. Although reports on the role of BMI1 in OS cells are not entirely consistent [22], we have demonstrated via in vivo and in vitro experiments that BMI1 promotes proliferation and metastasis of OS cells. In addition, we found that BMI1 directly binds to the promoter region of SIK1 in OS cells to inhibit transcription of SIK1. In the in vitro experiment, inhibition of SIK1 expression with siRNA in BMI1-deficient OS cells largely rescued the altered phenotype. Our findings strongly suggest that BMI1 can promote progression of OS by inhibiting the transcription of SIK1.

The major advance in the present study is our discovery of an important downstream direct target gene of BMI1 in OS. Thus far, there has been only one report on the molecular mechanism underlying the role of BMI1 in OS, which revealed that BMI1 promotes an invasive phenotype of OS by upregulating and activating MMP9 via NF-κB [13]. In recent years, additional downstream pathways of BMI1 have been identified in some other types of tumors, suggesting a broader and more complex role played by BMI1 in tumorigenesis and cancer progression [8, 16, 17, 48].

By using publicly available BMI1 ChIP-seq data from 3 cell lines (MCF-7 cell line, K562 cell line and GM12878 cell line), we predicted an enriched distribution of BMI1 binding sites from − 1 kb to + 1 kb relative to the TSS. We performed functional enrichment of BMI1 binding sites and identified many target genes. Among these, we focused on SIK1. Previous studies have shown that SIK1 is a tumor suppressor and is regulated by transcription factors in some cancers [26–29, 49]. We therefore speculated that BMI1 might be involved in regulation of SIK1 expression. Bioinformatic analysis of publicly available BMI1 ChIP-seq data for these 3 cell lines revealed a BMI1-binding peak at the promoter region of SIK1. Further, we found that mRNA expression of SIK1 was inversely correlated with that of BMI1 in OS cells. This finding suggests that BMI1 might somehow bind at the promoter region of SIK1 to contribute to inhibition of SIK1 in OS cells.

Recent work has shown that BMI1 and RING1B proteins, together with the accessory proteins CBX, HPH, rybp and pcgfs, form the core of PRC1 and catalyze histone modifications such as H2AK119ub (monoubiquitination of lysine 119 of H2A histone) [50]. PRC1-mediated monoubiquitination of H2AK119 is associated with gene repression [40, 42, 51]. Trimethylation of histone H3 at lysine 4 (H3K4me3) is necessary to prolong transcription [52], which is a modification of chromatin that marks transcriptional start points of active genes [53]. Zhou et al. [42] reported that BMI1 regulates a set of critical cardiogenic genes by directly binding to their regulatory regions, and knockdown of BMI1 results in a substantial loss of H2AK119ub and increase in H3K4me3 levels, which together are closely correlated with derepression of cardiogenic gene expression. Here, we performed ChIP-PCR assays and precipitated BMI1 at the predicted peak regions of BMI1 binding in the promoter region of SIK1. It was shown that RING1B and H2AK119ub were significantly reduced from the promoter region of SIK1 in BMI1-depleted OS cells compared with the control group. In addition, we found that precipitation of H3K4me3 in the promoter region of SIK1 of the PTC-209 group was much higher than in the control group. Our results strongly suggest that BMI1 forms a complex with RING1B, which promotes monoubiquitination of H2AK119 and decreases methylation of H3K4, thereby inhibiting transcription of SIK1.

Although SIK1 has been shown to be a tumor suppressor gene in multiple tumor types, no one has so far validated its role in OS cells. In our study, we inhibited expression of SIK1 with siRNA and observed that proliferation and migration of SIK1-depleted OS cells significantly increased, which demonstrates that SIK1 plays a role in tumor suppression. More interestingly, when we substantially knocked down BMI1 in OS cells, expression of SIK1 increased; Further knock-down of SIK1 restored proliferation and migration of BMI1-depleted OS cells. Our data strongly suggest that BMI1 promotes proliferation and metastasis of OS cells by repressing transcription of SIK1.

In this study, BMI1 was successfully immunoprecipitated from the SIK1 promoter region in OS cells, implying that BMI1 acts on SIK1 at the level of gene transcription. Knockdown of BMI1 significantly reduced levels of RING1B and H2AK119ub and increased levels of H3K4me3 in the promoter region of the SIK1 gene, thus promoting SIK1 transcription. These results indicate that BMI1 promotes H2AK119 monoubiquitination and inhibits H3K4 trimethylation by forming a complex with RING1B, which subsequently represses SIK1 transcription to promote development of OS.

## Conclusions

In conclusion, we have used in vivo and in vitro experiments to demonstrate the involvement of BMI1 in regulating proliferation and migration of OS cells. To our knowledge, this study is the first to show SIK1 to be a downstream direct target of BMI1. We have revealed that BMI1 exerts its cancer-promoting effect by mediating transcriptional repression of SIK1, which provides a potential therapeutic target for OS.

## Supplementary Information

**Additional file 1: Table S1.** Antibodies information.

**Additional file 2:** **Fig. S1.** The effect of BMI1 on OS cell proliferation, migration and invasion in vitro. **A** After OS cells were treated with si-NC or si-BMI1 1#, 2# for 48 h, the proliferative viability of OS cells was detected using CCK-8 assay. Sample size = 6. **B** Colony formation assays were used to detect the colony forming ability of OS cells after treated with si-NC or si-BMI1 1#, 2# for 48 h. Sample size = 3. **C** Quantification of **B**. **D** After treated with si-NC or si-BMI1 1#, 2# for 48 h, transwell assays were performed to determine the migration of OS cells. Sample size = 3. **E** Quantification of **D**. **F** Invasion assays were performed to detect the invasive ability of OS cells after treated with si-NC or si-BMI1 1#, 2# for 48 h. Sample size = 3. **G** Quantification of **F**. *p < 0.05, **p < 0.01, ***p < 0.001, compared with si-NC. **Fig. S2** BMI1 affects OS cells EMT in vivo and vitro. **A** Immunofluorescence staining of E-cadherin, N-cadherin, and Vimentin in control and PTC-209 treated group. Sample size = 3. Scale bar: 20 μm. **B** Quantification of **A**. **C** Realtime PCR analysis of Twist, Zeb1, Snail1, E-cadherin, N-cadherin, and Vimentin in 143B cells and U2OS cells treated with control (DMSO) or PTC-209. Sample size = 3. **D** Western blot evaluation of E-cadherin, N-cadherin and vimentin. Gapdh was used as an internal control. Sample size = 3. **E** Quantification of **D**. **F** Realtime PCR analysis of Twist, Zeb1, Snail1, E-cadherin, N-cadherin, and Vimentin in the metastatic nodules treated with control (DMSO) or PTC-209. *p < 0.05, **p < 0.01, ***p < 0.001, compared with control.

## Data Availability

The data sets used and/or analyzed during the current study are available from the corresponding author on reasonable request.
