# Supplementary material for: BMI1 promotes osteosarcoma proliferation and metastasis by repressing the transcription of SIK1
Source: Cancer Cell Int. 2022 Mar 27;22:136. doi: 10.1186/s12935-022-02552-8 (PMC8961961; doi:10.1186/s12935-022-02552-8)
Supplement: Supplementary file 1 — Additional file 1: Table S1. Antibodies information. [file 12935_2022_2552_MOESM1_ESM.docx]

**Supplementary Information**

**Table S1.** Antibodies information

| Antigen | Source | Company | Application | Dilution |
| --- | --- | --- | --- | --- |
| BMI1 | Rabbit | Proteintech | WB; IF; ChIP | 1:1000; 1:200 10μg/ChIP |
| Tubulin | Mouse | Beyotime | WB | 1:10000 |
| ub-Histone H2A(K119) | Rabbit | Cell Signal | ChIP | 10μg/ChIP |
| Ring1B | Mouse | Active Motif | ChIP | 5μg/ChIP |
| H3K4me3 | Rabbit | Milipore | ChIP | 5μg/ChIP |
| Ki67 | Rabbit | Abcam | IF | 1:200 |
| E-cadherin | Rabbit | Proteintech | IF; WB | 1:200; 1:1000 |
| N-cadherin | Rabbit | Proteintech | IF; WB | 1:200; 1:1000 |
| Vimentin | Rabbit | Cell Signal | IF; WB | 1:200; 1:1000 |
| Gapdh | Mouse | Proteintech | WB | 1:1000 |
|  |  |  |  |  |
