# Supplementary material for: BMI1 promotes osteosarcoma proliferation and metastasis by repressing the transcription of SIK1
Source: Cancer Cell Int. 2022 Mar 27;22:136. doi: 10.1186/s12935-022-02552-8 (PMC8961961; doi:10.1186/s12935-022-02552-8)
Supplement: Supplementary file 2 — Additional file 2: Fig. S1. The effect of BMI1 on OS cell proliferation, migration and invasion in vitro. A After OS cells were treated with si-NC or si-BMI1 1#, 2# for 48 h, the proliferative viability of OS cells was detected using CCK-8 assay. Sample size = 6. B Colony formation assays were used to detect the colony forming ability of OS cells after treated with si-NC or si-BMI1 1#, 2# for 48 h. Sample size = 3. C Quantification of B. D After treated with si-NC or si-BMI1 1#, 2# for 48 h, transwell assays were performed to determine the migration of OS cells. Sample size = 3. E Quantification of D. F Invasion assays were performed to detect the invasive ability of OS cells after treated with si-NC or si-BMI1 1#, 2# for 48 h. Sample size = 3. G Quantification of F. *p < 0.05, **p < 0.01, ***p < 0.001, compared with si-NC. Fig. S2 BMI1 affects OS cells EMT in vivo and vitro. A Immunofluorescence staining of E-cadherin, N-cadherin, and Vimentin in control and PTC-209 treated group. Sample size = 3. Scale bar: 20 μm. B Quantification of A. C Realtime PCR analysis of Twist, Zeb1, Snail1, E-cadherin, N-cadherin, and Vimentin in 143B cells and U2OS cells treated with control (DMSO) or PTC-209. Sample size = 3. D Western blot evaluation of E-cadherin, N-cadherin and vimentin. Gapdh was used as an internal control. Sample size = 3. E Quantification of D. F Realtime PCR analysis of Twist, Zeb1, Snail1, E-cadherin, N-cadherin, and Vimentin in the metastatic nodules treated with control (DMSO) or PTC-209. *p < 0.05, **p < 0.01, ***p < 0.001, compared with control. [file 12935_2022_2552_MOESM2_ESM.docx]

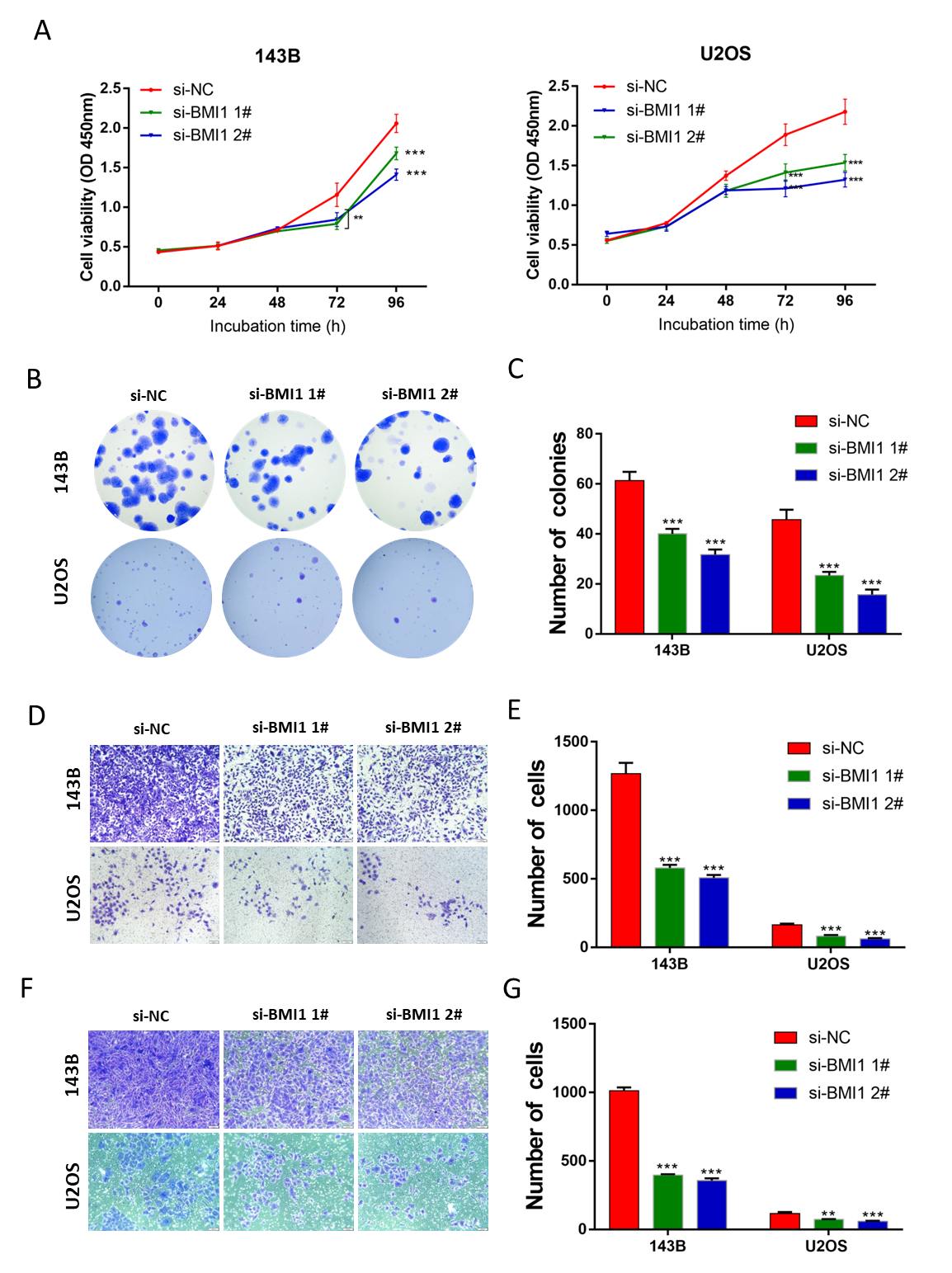


**Fig. S1** The effect of BMI1 on OS cell proliferation, migration and invasion in vitro. **A** After OS cells were treated with si-NC or si-BMI1 1#, 2# for 48 h, the proliferative viability of OS cells was detected using CCK-8 assay. Sample size = 6. **B** Colony formation assays were used to detect the colony forming ability of OS cells after treated with si-NC or si-BMI1 1#, 2# for 48 h. Sample size = 3. **C** Quantification of **B**. **D** After treated with si-NC or si-BMI1 1#, 2# for 48 h, transwell assays were performed to determine the migration of OS cells. Sample size = 3. **E** Quantification of **D**. **F** Invasion assays were performed to detect the invasive ability of OS cells after treated with si-NC or si-BMI1 1#, 2# for 48 h. Sample size = 3. **G** Quantification of **F**. *p < 0.05, **p < 0.01, ***p < 0.001, compared with si-NC.


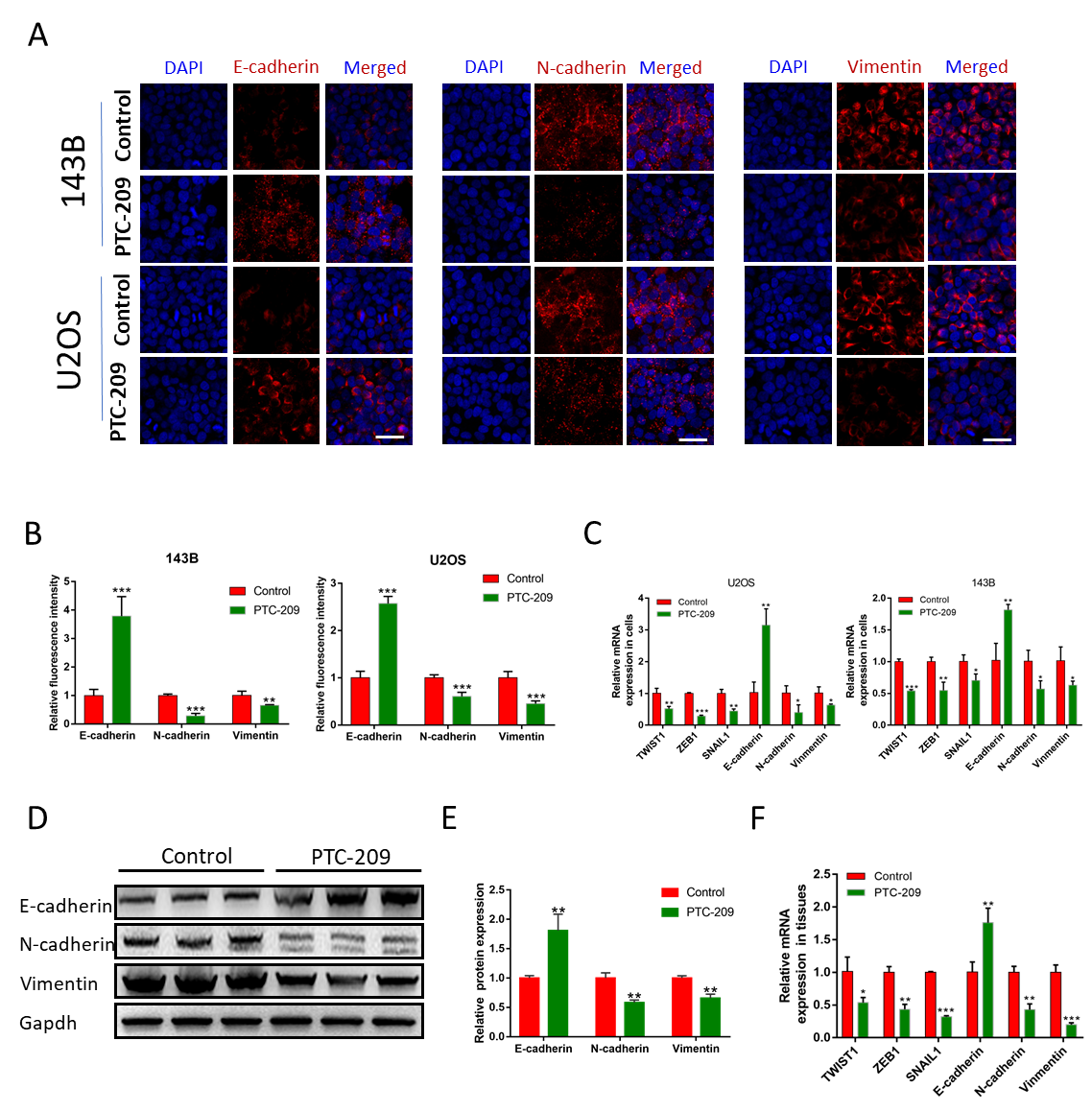


**Fig. S2** BMI1 affects OS cells EMT in vivo and vitro. **A** Immunofluorescence staining of E-cadherin, N-cadherin, and Vimentin in control and PTC-209 treated group. Sample size = 3. Scale bar: 20μm. **B** Quantification of **A**. **C** Realtime PCR analysis of Twist, Zeb1, Snail1, E-cadherin, N-cadherin, and Vimentin in 143B cells and U2OS cells treated with control (DMSO) or PTC-209. Sample size = 3. **D** Western blot evaluation of E-cadherin, N-cadherin and vimentin. Gapdh was used as an internal control. Sample size = 3. **E** Quantification of **D**. **F** Realtime PCR analysis of Twist, Zeb1, Snail1, E-cadherin, N-cadherin, and Vimentin in the metastatic nodules treated with control (DMSO) or PTC-209. *p < 0.05, **p < 0.01, ***p < 0.001, compared with control.
